# The Effects of Multiple Acute Turkesterone Doses on Indirect Measures of Hypertrophy and Metabolic Measures: A Preliminary Investigation

**DOI:** 10.3390/muscles3040031

**Published:** 2024-10-23

**Authors:** Dillon R. Harris, Tomas Chapman-Lopez, Steven B. Machek, Jeffery S. Forsse, Tracey Sulak, Leslee K. Funderburk

**Affiliations:** 1 Department of Health, Human Performance, and Recreation, Robbins College of Health and Human Sciences, Baylor University, Waco, TX 76706, USA; dillon_harris@tamu.edu (D.R.H.); tomas_chapman@baylor.edu (T.C.-L.); leslee_funderburk@baylor.edu (L.K.F.); 2 Department of Kinesiology and Sport Management, Texas A&M University, College Station, TX 77845, USA; 3 Department of Surgery, College of Medicine, University of Nebraska Medical Center, Omaha, NE 68105, USA; 4 Kinesiology Department, College of Health Sciences and Human Services, California State University, Monterey Bay, Seaside, CA 93955, USA; 5 Educational Psychology, Baylor University, Waco, TX 76798, USA; tracey_sulak@baylor.edu; 6 Human Sciences and Design, Baylor University, Waco, TX 76706, USA

**Keywords:** turkesterone, phytoecdysteroids, insulin-like growth factor-1 (IGF-1), metabolism, dietary supplement

## Abstract

Turkesterone is a naturally occurring plant steroid touted for its medicinal, pharmacological, and biological properties with no reported adverse side effects compared with traditional anabolic androgenic steroids (AAS). However, this ostensible enhancement to increase muscle protein synthesis and facilitate augmented thermogenesis remains undescribed despite uninformed and potentially haphazard consumption. To investigate whether turkesterone enhances insulin-like growth factor-1 (IGF-1) and resting metabolic rate (RMR), eleven apparently healthy males (23.3 ± 2.2) volunteered to participate in the present study with samples collected pre-, 3H post-, and 24H post-ingestion. Subsequent analyses failed to reveal any significant main condition, time, or interaction main effects for serum IGF-1, RMR, lipid, and carbohydrate metabolism (*p* > 0.05). However, non-significant serum IGF-1 concentrations increased with both turkesterone conditions and remained elevated when compared with placebo. Similarly, RMR remained elevated above baseline across the 3 h assessed. Although these data fail to fully support turkesterone as a potent anabolic supplement, nevertheless, our findings are foundational to persistently tease apart this supplement’s purported ergogenic effects and underscore its favorable hemodynamic and gastrointestinal tolerability profile. Future investigations should, therein, aim to assess turkesterone-mediated IGF-1 increases on long-term whole-muscle growth across several training sessions to further substantiate its efficacy on anabolism.

## 1. Introduction

Phytoecdysteroids (PE) are plant-derived analogues of the invertebrate molting hormones, playing a crucial role in synthesizing the deterrence against phytophagous (plant-eating) insects [1,2,3,4,5]. These naturally occurring polyhydroxylated ketosteroids are abundant with over 200 diverse species varying slightly in chemical structures [6]. Beyond their insecticidal properties, these arthropod hormones may influence plant growth, stress responses, and interact with various organisms within the ecosystem, prominently being found in plants such as *Ajuga turkenstanica*, *Rhaponticum carthamoides*, and *Schisandra chinensis* [7,8]. Historically, these compounds have garnered interest for their potential medical applications or as dietary supplements through their potential adaptogenic (propensity to help respond to stress, anxiety, fatigue, and overall wellbeing) and anabolic qualities [9]. Recent research has suggested a range of ergogenic and therapeutic effects, although detailed studies on their safety and efficacy are ongoing [10]. Despite bearing structural similarities to vertebrate steroid hormones (e.g., testosterone, estrogen, progesterone, and cortisol), PEs incidentally do not bind to cytosolic steroid receptors; rather, they primarily exert their ergogenic effect through various metabolic pathways such as protein synthesis, as well as lipid and carbohydrate metabolism [1]. Among these compounds, turkesterone—a derivative of *Ajuga turkenstanica*—has emerged as a particularly notable PE due to its potential benefits in enhancing muscle recovery, growth, and thermogenesis (fat burning) [1].

Turkesterone also appears to be a more potent anabolic agent relative to other PEs, surpassing cyasterone and 20-hydroxyecdysone (20E) in effectiveness when compared directly [11,12,13,14]. This anabolic effect in muscle was originally noted by Syrov in 1976, in which 0.5 mg/kg of 20E, turkesterone, and other PEs were administered daily for 10 days to castrated rats [15]. The study revealed significant increases in muscle mass, liver, and total protein content, suggesting pronounced anabolic activity. Another investigation by Syrov et al. [16] further demonstrated that turkesterone’s latent anabolic effects were comparable to those of traditional androgenic anabolic steroids (AAS) in terms of acute muscle protein synthesis (MPS) through increased muscle weight and total protein level. Although AAS-mediated anabolic effects are facilitated through androgen receptor activation and subsequent nuclear translocation, PE mechanisms of action are not fully understood; they are currently believed to impact signal transduction pathways through molecules binding to various receptor interactions [1]. Specifically, MPS and muscle protein breakdown (MPB) are allosterically controlled by the phosphoinositide 3-kinase (PI3K)/Akt (protein kinase B)/mammalian target of rapamycin (mTOR) pathway, which is a predominant mediator of load–skeletal muscle hypertrophy [17]. Turkesterone is therein postulated to have a signaling effect via membrane-bound estrogen receptor beta (ER-β) binding and subsequent increases in insulin-like growth factor-1 (IGF-1) transcription, which binds to the insulin receptors to facilitate PI3K/Akt/mTOR pathway activation [18]. Additionally, IGF-1 is a well-established hormone that binds to insulin receptors and improves translational efficiency via the PI3K-Akt-mTORC1 pathway [4]. A recent in vitro study further reinforces turkesterone to augment this postulated anabolic environment through increased circulating IGF-1 concentrations and reducing myostatin gene expression [6]. Lawrence et al. [4] corroborated this effect in an investigation in aged rats supplemented orally with turkesterone, observing increased protein synthesis, cross-sectional skeletal muscle area, and mitochondrial biogenesis. Consequently, research also suggests that these arthropod compounds may favorably effect lipid and carbohydrate metabolism through altering changes in energy substrate utilization, which could further support its anecdotal role in enhanced body composition via augmented resting metabolic rate (RMR) [19].

However, there is a significant scarcity of the literature examining the pharmacological impacts on human physiology and metabolism [14]. Anthony et al. [20] reported that PEs have a low oral bioavailability, so an effective dose–response to potentially support body composition remains uninvestigated in humans. Additionally, there is no evidence regarding individual tolerance of turkesterone during oral digestion. Addressing these gaps is crucial to understand the dose–response relationship and digestibility of turkesterone, significantly impacting its potential health-associated applications and underscoring the possible risk to recreational users in the broad community. Therefore, the primary purpose of this study is to investigate the hypothesis that turkesterone administration in varying doses creates an increase in hypertrophy-associated serum markers and RMR with favorable oral digestibility tolerance.

## 2. Materials and Methods

### 2.1. Experimental Approach

In this randomized, single-blinded, placebo-controlled cross-over experimental design, participants visited the laboratory on four occasions, undergoing a series of analyses on visits 2–4, including a digestion questionnaire, venipuncture, resting metabolic rate (RMR) assessment, heart rate (HR) and blood pressure (BP; systolic [SBP] and diastolic [DBP] blood pressure) determination, and total body water (TBW) measurement. The laboratory was climate controlled throughout the duration of this study with the following average recorded variables: temperature = 21.9 ± 1.4 °C; humidity = 57.6 ± 9.3%; and barometric pressure = 752 ± 5.4 mmHg. On the first visit (barring consent and meeting inclusion criteria), the participant received a comprehensive verbal outline of all the subsequent procedures, as well as body composition analysis via dual energy X-ray absorptiometry (DEXA) for body fat and lean mass determination. Furthermore, participants provided a 48-hour dietary intake prior to each visit through the MyFitnessPal application (Under Armor, San Francisco, CA, USA) [21,22,23] and underwent the first testing sequence under randomized conditions on visit 2–4. All testing sessions occurred between 6:00 am and 10:00 am, with most assessments beginning at 8:00 am.

### 2.2. Participants

Per American College of Sports Medicine (ACSM), eleven apparently healthy, recreationally active (≥3 days/week, over the last 6 months) males between the ages of 18–35 years volunteered and completed this study [24]. Participant descriptive data are depicted in Table 1. All were subject to an initial familiarization and screening visit, which included a comprehensive medical questionnaire and consent form. Briefly, only participants considered as either low or moderate risk as outlined by the ACSM and/or had any dietary restrictions or supplement regimens (i.e., taking anabolic androgenic steroids, selective androgen receptor modulators, creatine, and/or any other ergogenic aids) that could be performance enhancing were not allowed to participate [25]. All eligible participants signed university-approved informed consent documents and approval was granted by the Institutional Review Board for Human Subjects in Research of Baylor University (protocol # 1977690, 19 January 2023). In addition, all experimental procedures involved in this study conformed to the ethical consideration of the Declaration of Helsinki.

### 2.3. Anthropometrics Analyses

During the entry/familiarization visit, total body mass (kg) was determined on a standard dual-beam balance scale (Detecto, Bridgeview, IL, USA). Furthermore, percent body fat, fat mass, lean muscle mass, and bone mineral density were assessed using DEXA (Hologic Discovery Series W, Waltham, MA, USA). Quality control calibration procedures were performed on a spine phantom (Hologic X-CALIBER Model DPA/QDR-1 anthropometric spine phantom) and a density step calibration phantom prior to each testing session.

### 2.4. Venipuncture

Following an 8 h fast, venous blood sampling was obtained using 10 mL vacutainer tubes from a 22-gauge phlebotomy needle inserted into the antecubital vein. Blood samples were allowed to stand at room temperature for 10–15 min and then subsequently centrifuged at 2500 rpm for 15 min. The serum was removed and frozen at −80 °C for later analysis. One hundred and sixty-five blood samples were obtained throughout this study. The blood samples were collected prior to each condition (PRE), 1 h post- (POST1H), 3 h post- (POST3H), and 24 h post-supplement ingestion (POST24H).

### 2.5. Supplementation Protocol

During each visit, subjects consumed a singular dose of either condition (1000 mg [with 1000 mg inert cellulose placebo] and 2000 mg) of turkesterone (TURK; Double Wood Supplements, Sharon Hill, PA, USA) or 2000 mg inert cellulose placebo (PLA; NutriCology, South Salt Lake, UT, USA) in the morning an hour prior to testing, wherein these dosages were chosen accordingly as most manufacturers recommend 1000 mg/day while suggesting to not exceed 2000 mg/day. Although the half-life of turkesterone is not fully understood, it is purported to share a similar half-life with ecdysterone (20HE; another PE derivative) between 4 and 9 h; therefore, to ensure proper washout of the supplement participants were required to wait approximately ≥7 days between each experimental condition [26,27]. Supplement preparation and distribution was performed in a single-blind and counterbalanced manner, whereby each participant was asked to close their eyes before consuming the experimental condition. Additionally, each supplemental condition was placed into a nontransparent paper cup to further blind the participant, ingested with four ounces of water, and was witnessed by research personnel for all testing sessions.

### 2.6. Serum Target Analysis

Serum IGF-1 concentrations were assessed via commercially available enzyme-linked immunosorbent assay (ELISA) kits (MyBiosource, San Diego, CA, USA). Sample absorbance was read at a wavelength of 450 nm and unknown concentrations determined by linear regression against known standard curves using commercial software (Microplate Manager Software 5.2.1, Bio-Rad, Hercules, CA, USA). The average intra-assay and inter-assay coefficients of variation (CV%) were 8.91% and 10.12%, respectively.

### 2.7. Resting Metabolic Rate Analysis

To determine turkesterone’s effect on metabolism, the commercially available metabolic cart (Parvo Medics TrueOne 2400, Salt Lake City, UT, USA) was used to calculate and assess RMR and respiratory exchange ratio (RER). This metabolic cart has been validated to provide reliable measurements of oxygen consumption (VO_2_) and carbon dioxide expiration (VCO_2_) [28]. An empirically validated facemask was placed over the participant’s face and attached to the flowmeter to collect sample expired air as per manufacturer instructions and flow rate was established within the first 5 min of sampling [29,30]. To determine RMR, at sessions 2–4, respiratory gases (VO_2_ and VCO_2_) were measured continuously using an integrated respiratory gas analysis system adapted from the methods described by Alcantrara et al. [31]. Briefly, the participants were asked to lie still while refraining from using electronic devices during the assessment. Moreover, each RMR assessment lasted 20 min to determine any changes from baseline across a 3 h period with the first 5 min being discarded as to ensure each participant reached a resting state as well as limiting physical activity between assessments [30].

### 2.8. Gastrointestinal and Hemodynamic Assessments

**Figure 1:**
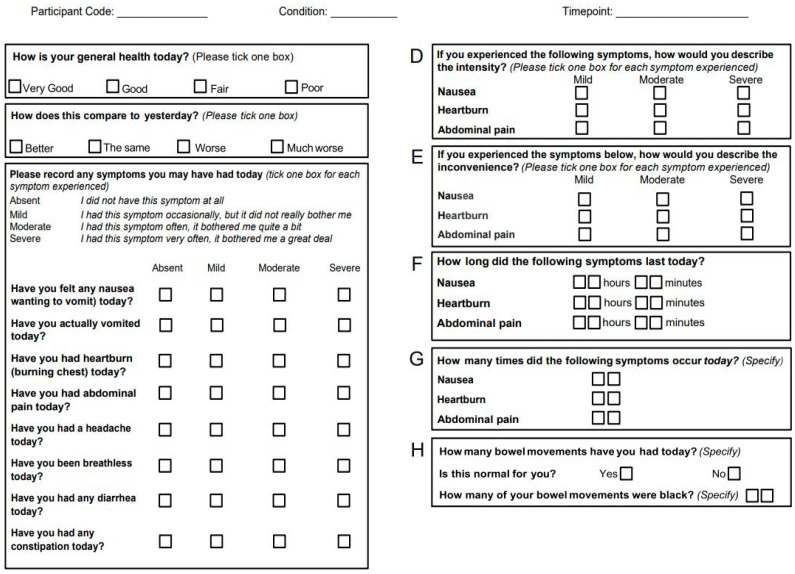
One-page symptom questionnaire to assess gastrointestinal adverse effects after oral turkesterone supplementation.

To generate a better understanding of turkesterone’s influence on metabolic and cardiovascular parameters, participants were asked to report their GI distress symptoms by answering a comprehensive survey adapted from Periera et al. [32]. Briefly, this one-page symptom questionnaire asks several questions related to GI distress, including nausea, vomiting, heartburn, abdominal pain, etc. (see Figure 1). Furthermore, participants were asked to rest in a seated position for five minutes and fitted with a blood pressure cuff and associated sphygmomanometer to assess BP. HR was manually palpated and subsequently recorded during each time point (PRE, POST1H, POST3H, and POST24H).

### 2.9. Statistical Analyses

A prior α-priori power analysis was completed using studies of similar methodological design to establish the adequate number of participants, wherein it determined nine participants were necessary to achieve an anticipated η_p_^2^ = 0.40, and power (1-ß) = 0.80 at α = 0.05. [33,34,35]. All variables were tested for normality and homogeneity of variance using the Shapiro–Wilk test and Levene’s test of homogeneity of variance, including Mauchly’s test of sphericity before continuing subsequent statistical analysis. Serum IGF-1, GI questionnaire, RMR, BP, and HR were assessed using separate two-way analysis of variance (ANOVA) for condition (1000 mg, 2000 mg, & placebo) × time (PRE, POST3H, POST24H) with repeated measures. Body fat (%), lean mass, and bone mineral density as well as 48 h dietary recall data (total kcal, macronutrients, and fiber [all in g/kg bodyweight]) were assessed via one-way ANOVA with repeated measures for each supplement condition. If a subsequently significant interaction was found, pairwise comparisons with a Bonferroni adjustment for alpha inflation was used to compare group means. The index of effect size was partial Eta squared (η_p_^2^), which estimates the proportion of variance in the dependent variable that can be explained by the independent variable. Partial Eta squared effect sizes were determined to be: weak = 0.17, medium = 0.24, strong = 0.51, very strong = 0.70 [36]. Additionally, Kendall’s W coefficient of concordance was used as an estimate of agreement for nonparametric data, whereby 0–0.20 = slight agreement, 0.20–0.39 = fair agreement, 0.40–0.59 = moderate agreement, 0.60–0.79 = substantial agreement, and >0.80 = almost perfect agreement [37]. For all statistical analyses, an alpha level of <0.05 was adopted throughout and values reported as means ± standard deviations (SD). Confidence intervals (CI) for significant comparisons were reported as 95% CI (lower bound, upper bound).

## 3. Results

### 3.1. Dietary Analysis

Analyses failed to reveal any significant main effects for total kilocalorie nor any macronutrient (carbohydrates, protein, or fat) intake across conditions. All dietary mean ± SD, significance levels, effect sizes, and CIs are reported in Table 2.

### 3.2. Resting Metabolic Rate Analysis

**Figure 2:**
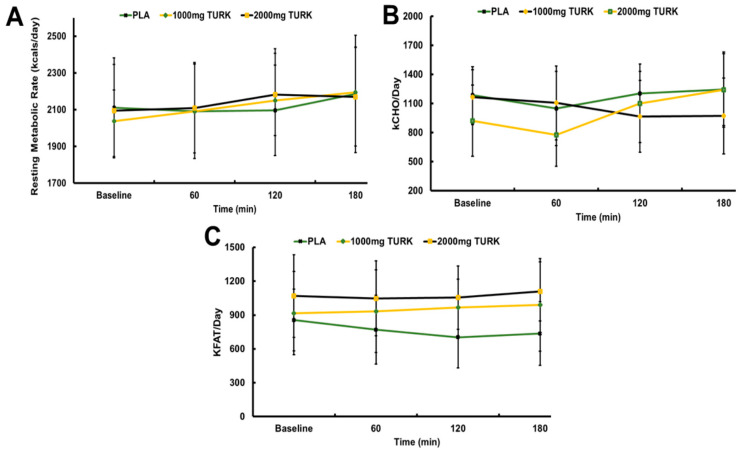
Two-way repeated measures ANOVA for (**A**) changes in resting metabolic rate (RMR) from baseline to 3 h post-ingestion across each condition, (**B**) changes in carbohydrate utilization from baseline to 3 h post-ingestion across each condition, and (**C**) changes in fat utilization from baseline to 3 h post-ingestion between each condition. Data are expressed as mean ± SD.

Analyses failed to detect any significant main supplement condition (*p* = 0.866; η_p_^2^ = 0.031) nor time (*p* = 0.903; η_p_^2^ = 0.065) effects for kcals/day, as well as no significant condition × time (*p* = 0.309; η_p_^2^ = 0.659) interaction effect (see Figure 2A). When comparing carbohydrate oxidation (see Figure 2B), there was no significant difference between conditions (*p* = 0.546; η_p_^2^ = 0.126). Moreover, there were no significant time (*p* = 0.328; η_p_^2^ = 0.335) main effect nor condition × time (*p* = 0.847; η_p_^2^ = 0.329) interaction effects. Analysis of fat oxidation (see Figure 2C) failed to reveal any main condition (*p* = 0.152; η_p_^2^ = 0.342) nor time (*p* = 0.700; η_p_^2^ = 0.155) main effects, or a condition × time (*p* = 0.998; η_p_^2^ = 0.069) interaction effect.

### 3.3. IGF-1 Serum Concentrations

All raw serum IGF-1 data can be found in Table 3. Baseline serum IGF-1 was not different between supplement conditions (*p* = 0.180; η_p_^2^ = 0.193). Analyses further failed to reveal any significant condition (*p* = 0.178; η_p_^2^ = 0.438), time (*p* = 0.227; η_p_^2^ = 0.390), nor any condition × time (*p* = 0.547; η_p_^2^ = 0.211) effects for serum IGF-1. Incidentally, 24H post-serum IGF-1 failed normality assumptions, but a nonparametric Friedman’s ANOVA further confirmed that no significant condition-specific differences (χ^2^ = 4.333, *p* = 0.115) were present.

### 3.4. Gastrointestinal Distress and Hemodynamic Response

None of the participants reported the presence of any symptoms throughout the course of this study between either turkesterone condition and placebo. There were no main condition (*p* = 0.479; η_p_^2^ = 0.100) nor time (*p* = 0.539; η_p_^2^ = 0.102) main effects as well as no condition × time interaction effects (*p* = 0.469; η_p_^2^ = 0.122) for heart rate. For systolic blood pressure (SBP), analyses failed to reveal any significant main condition (*p* = 0.636; η_p_^2^ = 0.063) nor time (*p* = 0.454; η_p_^2^ = 0.119) effects, as well as no condition × time interaction effect (*p* = 0.123; η_p_^2^ = 0.194). Similarly, diastolic blood pressure had no significant main supplement (*p* = 0.427; η_p_^2^ = 0.114) nor any time (*p* = 0.912; η_p_^2^ = 0.033) effects, without a significant condition × time effect (*p* = 0.932; η_p_^2^ = 0.050). Lastly, no differences were seen in TBW at any timepoints. See Table 4 for a more detailed description of all hemodynamic data.

## 4. Discussion

These data are the first to report on the purported ergogenic efficacy of turkesterone relative to several body composition-associated variables, including growth-associated IGF-1 and RMR [38]. Our findings nevertheless failed to support the predetermined hypotheses, predominantly indicating that the included turkesterone doses were unable to significantly improve any of the aforementioned metrics when compared with an inert placebo in recreationally active males, and neither turkesterone condition was ultimately able to statistically increase serum IGF-1 concentrations relative to placebo. It is nonetheless imperative to note that despite our equivocal findings, both turkesterone groups demonstrated higher mean concentrations at 3 h post-ingestion relative to the placebo and these remained elevated at 24 h. Other investigations by Parr et al. [39] and Tashmukhamedova et al. [40] saw that PE-administered male rats significantly increased serum IGF-1 concentrations and associated muscle weight relative to placebo, suggesting the potential to facilitate a favorable anabolic environment [39,40]. While the human literature in this area is strikingly sparse and with mixed results, it is worth noting that Isenmann et al. [41] observed hypertrophic effects similar to previous in vivo investigations in (human) individuals supplemented with 20E-containing products while undergoing a 10-week resistance training program [41]. It is possible that our equivocal findings may be due to the low bioavailability prevent in most PEs and that most available turkesterone products are commonly standardized to 10% of specifically isolated turkesterone from ajuga turkenstanica [26]. Moreover, alterative PE like 20E demonstrates a relatively low half-life (approximately 3 h) before being completely eliminated via urine [27], ultimately suggesting that higher turkesterone supplement dosing regimens may be required to elicit the anecdotally supported hypertrophic outcomes reported in humans.

Other dietary supplements (caffeine, yohimbine, and several others) are popular weight loss aids, partially associated with their ability to antagonize adenosine receptor binding; this results in elevated norepinephrine (NE) concentrations that concomitantly mediate increased glycolytic, lipolytic, and general metabolic rates [42,43]. Previous research on other PE compounds has supported this effect, demonstrating effects such as maintained serum NE concentrations, elevated fatty acid oxidation, and overall stress reductions [44,45]. Conversely to any of the aforementioned compounds, our findings failed to support turkesterone’s influence on any adrenergic signaling-relevant mechanisms. Although not statistically significant, the current study saw that RMR was increased by 2.7%, 5.6%, and 7.8% in the 1000 mg TURK condition, Similarly, 2000 mg TURK condition only increased RMR by 0.7%, 4.2%, and 3.6% above baseline at 60, 120, and 180 min post-ingestion, respectively. PLA decreased by 0.9% and 0.7% below baseline at 60 and 120 min post-ingestion, respectively, and, at 180 min, it increased 3.6% above baseline. Wilborn et al. [46] examined the effects of methoxyisoflavone, 20E, and sulfo-polysaccharide supplementation, similarly failing to find changes in catecholamine synthesis-associated cortisol across an 8-week resistance training protocol [47]. Nevertheless, the data are mixed on these metabolic effects and it thus remains possible that turkesterone supplementation in our investigation may have decreased cortisol and catecholamines, resulting in the equivocally observed RMR and substrate utilization results [10]. In the current study, all supplement conditions saw a decrease at 60 min post-ingestion (11.7% [PLA], 5.1% [1000 mg TURK], and 15.6% [2000 mg TURK], respectively). The 1000 mg TURK condition continued to decrease below baseline by 17.1% and 16.6% at 120 and 180 min post-ingestion. Conversely, both 2000 mg TURK and PLA saw increases above baseline by 19.5% and 1.56% and 19.5% and 4.85% at 120 and 180 min post-supplement ingestion. Although not statistically significant, the 1000 mg TURK group saw—albeit not statistically significant— increased fat utilization by 1.85%, 5.34%, and 7.96%. Both PLA and 2000 mg TURK, however, saw decreased fat utilization at each timepoint; this necessitates additional research to corroborate our data before more definitive statements can be made.

The secondary purpose in our study was to examine the effects of multiple turkesterone doses on resting hemodynamic variables and gastrointestinal tolerability. Briefly, acute doses of either 1000 mg nor 2000 mg TURK significantly altered heart rate, (systolic or diastolic) blood pressure, nor gastrointestinal distress symptoms. Although no changes were observed in either heart rate or blood pressure, high antioxidant-associated compound-containing diets may ultimately reduce blood pressure and cardiovascular disease [48]. Therefore, longitudinal dosing might be necessary to elicit these positive impacts on cardiovascular outcomes. Another plausible explanation is the aforementioned hypothetical turkesterone-mediated reduction in catecholamine excretion; this would facilitate augmented peripheral vasodilation and concomitantly attenuated cardiac stress. These data are nonetheless sparse overall and nonexistent in human demographics, so any speculations are contentious at best.

## 5. Limitations and Future Directions

Although the current findings provide a novel glimpse into the potential relationship with turkesterone supplementation on indirect measures of hypertrophy and metabolism, it was limited by several design characteristics. Specifically, our acute study design cannot be sensibly extrapolated to longitudinal outcomes as more commonly described in rodent models [4,11,13,15,16]. Although participants were statistically consistent with regards to nutrient intake across their visits, diet was not standardized and could have affected variables such as RMR or substrate utilization. Furthermore, future research would benefit from larger sample sizes and more precise measurements of hypertrophic outcomes. While we were adequately powered, it should not be discounted that this is the first human investigation on turkesterone supplementation and that our power analyses were predicated on other PE. Moreover, IGF-1 was an empirically supported hypertrophic-associated serum biomarker, but additional targets such as the previously mentioned PI3K/Akt/mTOR proteins, fractional protein synthesis analysis, and/or ultrasound-based cross sectional area measurement would provide more relevant conclusions [49,50,51,52]. Finally, future investigations should employ an exercise program to determine turkesterone’s potentially permissive role in adaptation. One of the potential reasons for the mixed hypertrophic outcomes in prior trials may be differences in training regimen and length. Namely, Isennman et al. [41] and Wilborn et al. [46] saw conflicting outcomes, whereby the former saw hypertrophic enhancement with only a two-week longer resistance training trial (10 weeks versus 8 weeks). This may therefore suggest that turkesterone facilitates chronic exercise adaptation more so over functioning acutely. We nonetheless choose an acute design due to the novel implementation of this supplement in human participants, seeking to eliminate exercise as an extraneous variable and fostering a foundation for future research.

## 6. Conclusions

Our data demonstrate that neither 1000 mg nor 2000 mg of turkesterone was able to significantly increase RMR and also failed to alter IGF-1, RMR, and substrate utilization relative to placebo. Furthermore, acute supplementation did not adversely impact resting heart rate, blood pressure, or subjective gastrointestinal tolerability. Despite these equivocal findings, the latter is especially relevant considering turkesterone’s recreational prevalence; doses up to 2000 mg are well tolerated, posing little-to-no risk to general population consumption with acute consumption. Thus, future research can expand upon these doses with complementary interventions and/or possibly increasing doses to find the appropriate dose–response. These data, nevertheless, are the first to elucidate this supplement’s potential ergogenic role towards enhancing human body composition. Future research should thereby build upon our findings to exhaustively tease out turkesterone’s potential before making any definitive recommendations.

## Figures and Tables

**Table 1:** Participant descriptive data including anthropometrics and body composition *.

Participants (*n* = 11)	Mean ± SD
Age (y)	23.3 ± 2.2
Height (cm)	179.3 ± 7.9
Weight (kg)	91 ± 18.5
Body Fat (%)	18.6 ± 7.1
Lean Mass (kg)	71.16 ± 10.97

* All data are reported as mean ± SD.

**Table 2:** Average 48 h intake across experimental conditions one week apart *.

Mean ± SD, 95% CI (LB, UB)	PLA	1000 mg	2000 mg	*p*-Value	η_p_^2^
Carbohydrate (g∙kg^−1^)	2.9 ± 0.8 (2.3, 3.5)	2.8 ± 1.0 (2.3, 3.9)	3.1 ± 0.9 (2.5, 3.8)	0.747	0.079
Fat (g∙kg^−1^)	1.2 ± 0.4 (0.9, 1.5)	1.0 ± 0.4 (0.8, 1.3)	1.0 ± 0.3 (0.8, 1.3)	0.160	0.160
Protein (g∙kg^−1^)	1.9 ± 0.6 (1.5, 2.4)	1.6 ± 0.4 (1.3, 2.0)	1.7 ± 0.7 (1.2, 2.2)	0.421	0.083
Total kcal	2679 ± 727 (2231, 3161)	2486 ± 938 (1832, 3046)	2535 ± 318 (2262, 2716)	0.356	0.098

* CI = confidence interval; LB = lower bound; UB = upper bound. ¡Macronutrients were standardized to body mass (kg). All data are reported as mean ± SD. There were no statistical differences between condition intakes for any dietary parameters (*p* ≥ 0.05).

**Table 3:** Serum IGF-1 concentrations across all supplement and time combinations *.

Mean ± SD IGF-1 (ng/mL^−1^)	1000 mg TURK	2000 mg TURK	PLA
Pre	80.6 ± 32.9	89.4 ± 27.9	72.1 ± 30.8
3H Post	135.3 ± 53.7	147.9 ± 42.6	72.9 ± 33.3
24H Post	112.2 ± 58.8	139.2 ± 39	70.8 ± 28.2

* IGF-1 = insulin-like growth factor-1; PLA = placebo; TURK = turkesterone. All data are reported as mean ± SD. There were no statistical differences between condition intakes for IGF-1 serum concentrations across timepoints (*p* ≥ 0.05).

**Table 4:** Effects of turkesterone on hemodynamic response in apparently healthy males *.

Mean ± SD	1000 mg TURK			2000 mg TURK			PLA		
	PRE	3HPOST	24HPOST	PRE	3HPOST	24HPOST	PRE	3HPOST	24HPOST
HR (bpm)	63.3 ± 11.7	63.6 ± 12.9	67.8 ± 14.9	66.6 ± 7.6	62.9 ± 6.5	63.9 ± 10.3	63.4 ± 9.4	64.1 ± 9	66.2 ± 8.6
SBP (mmhg)	121 ± 4.7	122.1 ± 6.7	118.9 ± 3.7	123.7 ± 4.1	122.8 ± 3.6	123.4 ± 3.9	120.6 ± 4.7	121 ± 3.8	122.4 ± 8.6
DBP (mmhg)	77.3 ± 4.2	78.2 ± 4.7	76.7 ± 3.1	79.7 ± 3.3	79.1 ± 4.9	78.7 ± 2.6	77 ± 5	78 ± 4.9	78.1 ± 3.7

* bpm = beats per minute; DBP = diastolic blood pressure; HR = heart rate; PLA = placebo; SBP = systolic blood pressure; TURK = turkesterone. All data are reported as mean ± SD. There were no statistical differences between condition intakes for any hemodynamic responses (*p* ≥ 0.05).

## Data Availability

All the data and materials associated with the findings stated in the results of this manuscript are within the manuscript.
